# Quantifying the Impact of Expanded Age Group Campaigns for Polio Eradication

**DOI:** 10.1371/journal.pone.0113538

**Published:** 2014-12-01

**Authors:** Bradley G. Wagner, Matthew R. Behrend, Daniel J. Klein, Alexander M. Upfill-Brown, Philip A. Eckhoff, Hao Hu

**Affiliations:** Institute for Disease Modeling, Bellevue, Washington, United States of America; University of Waterloo, Canada

## Abstract

A priority of the Global Polio Eradication Initiative (GPEI) 2013–2018 strategic plan is to evaluate the potential impact on polio eradication resulting from expanding one or more Supplementary Immunization Activities (SIAs) to children beyond age five-years in polio endemic countries. It has been hypothesized that such expanded age group (EAG) campaigns could accelerate polio eradication by eliminating immunity gaps in older children that may have resulted from past periods of low vaccination coverage. Using an individual-based mathematical model, we quantified the impact of EAG campaigns in terms of probability of elimination, reduction in polio transmission and age stratified immunity levels. The model was specifically calibrated to seroprevalence data from a polio-endemic region: Zaria, Nigeria. We compared the impact of EAG campaigns, which depend only on age, to more targeted interventions which focus on reaching missed populations. We found that EAG campaigns would not significantly improve prospects for polio eradication; the probability of elimination increased by 8% (from 24% at baseline to 32%) when expanding three annual SIAs to 5–14 year old children and by 18% when expanding all six annual SIAs. In contrast, expanding only two of the annual SIAs to target hard-to-reach populations at modest vaccination coverage—representing less than one tenth of additional vaccinations required for the six SIA EAG scenario—increased the probability of elimination by 55%. Implementation of EAG campaigns in polio endemic regions would not improve prospects for eradication. In endemic areas, vaccination campaigns which do not target missed populations will not benefit polio eradication efforts.

## Introduction

In May 2012, the World Health Assembly declared the eradication of polio a “programmatic emergency for global public health” and called for a comprehensive polio endgame strategy [1]. Major progress has been made since the founding of the Global Polio Eradication Initiative (GPEI) Program in 1988 [2]. Polio is now endemic in only three countries as of 2014, and case counts are reaching historic lows. For the first time ever, the world is on the verge of eradicating wild poliovirus. Achievement of this goal will benefit future generations and allow the more than one billion dollar annual budget for GPEI to be directed towards other health and development goals.

Population immunity against polio infection and paralysis can be gained directly from Routine Immunization (RI) or Supplemental Immunization Activity (SIA) campaigns as well as from secondary transmission of vaccine virus or prior wild poliovirus infection. Low case-to-infection ratio, long virus shedding periods, and the high transmissibility of wild virus necessitate high vaccination coverage in endemic countries, as well as prompt elimination of population immunity gaps [3], [4]. This approach prevents the virus from reaching susceptible pockets large enough to sustain circulation.

The delivery of Oral Polio Vaccine (OPV) to children less than 5 years of age is standard practice in most SIA campaigns [1]. This age group bears a majority of the morbidity and mortality of polio. Recent program strategy discussions have asked whether expansion of the target age group beyond five years will increase the impact of eradication efforts. Past periods of low vaccination coverage may have contributed to low immunity levels in older children [5]. If such an immunity gap exists, vaccinating older children would be of benefit. Beyond epidemiological factors there may be practical programmatic reasons for employing expanded age group (EAG) campaigns. Such campaigns may enable health workers to vaccinate children without being certain of their ages. This may improve coverage among younger children, but it is unclear whether success of this kind can be quantified, or if it is possible to verify whether the additional children reached were previously vaccinated.

The cost of EAG campaigns is well beyond the status quo. Higher expenses are due to additional vaccine requirements as well as the need to recruit, train, and organize additional vaccinators for synchronized SIA campaigns [6]. These costs make it imperative to quantify the reduction in virus transmission, the effect of improved coverage, and the additional benefit of passive immunization before large-scale implementation of EAG campaigns.

## Methods

In this work we quantify the potential benefit of EAG campaigns to polio eradication. We constructed a mathematical model of polio immunity and transmission calibrated to Zaria, Nigeria seroprevalence survey data [7]. The model is individual-based, with transmission of poliovirus occurring through virus shedding and acquisition among individuals and their communities. For each individual, key aspects related to the research question are included, such as dynamics of the immune response to OPV [8], [9], the relationship of mucosal immunity to probability of infection and subsequent virus shedding quantity [3], [10]–[13], transmission of wild poliovirus and vaccines [14]–[16], waning of mucosal and humoral immunity [17]–[21], and accessibility in campaigns.

Data were parameterized in three ways throughout this work. Firstly, parameterization of immune priming and boosting were set through the previous calibration of Behrend *et al.* to vaccine challenge data [3]. These involve the linear parameters for vaccine boosting and priming (see Table S1 in File S1), rate of virus neutralization by antibodies, per virion infectivity of vaccine viruses, and viral interference for coinfection with multiple serotypes. Data on decay of humoral antibodies as well as duration and intensity of viral shed and relationship to immunity were established (see Table S1 in File S1) through cross sectional and longitudinal data [12]. Decay of maternal antibodies is also included (Figure S3 in in File S1). The second set of parameters was established through calibration to age-stratified seroprevalence from the 2007 Zaria Nigeria serosurvey [7]. These are the size of vaccine inaccessible populations, the historical coverage of SIAs, the per virion infectivity of wild-virus, and the strength of population level mixing. The latter may be thought of as incorporating density, sanitation and survivability of the pathogen in the environment. Thirdly, as the rate of decay of mucosal antibodies is not practically measurable, we performed our population calibration under the assumptions of fast and slow waning mucosal antibodies, to establish the uncertainty of our results to this assumption. Historical routine immunization coverage was not parameterized, but was set at 30% consistent with existing data for Northern Nigeria. Parameter definitions and values are given in Table S1 in File S1.

The intrahost model includes immunity, infection, and other individual state variables. Immunity is tracked in both the humoral and mucosal compartments of the immune system. Humoral immunity protects infected individuals from paralysis if they are seropositive (neutralizing antibody titer is typically larger than 1∶8 serum dilution). It is tracked on a continuum to compare population immunity to population seroprevalence surveys for calibration.

**Figure 1:**
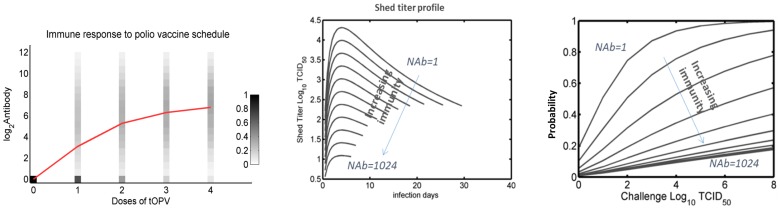
Behavior of the polio model. (**A**) Distribution of mucosal immunity by number of OPV doses given to a previously naïve individual. The depth axis in figure represents the probability distribution of antibody titer in the stochastic model and the red line represents the population mean of the antibody titer resulting from the distribution. (**B**) Shedding as a function of initial mucosal immunity and time since infection. (**C**) Probability of infection with different immunity levels and challenge doses.

Figure 1(A) shows the dynamics of immunity response to OPV doses in the model. The full details of the intrahost model are described in section 1.1 in File S1. Figure 1(B) shows neutralizing antibodies reducing the shedding titer expressed in terms of TCID50; the amount of virus required for a 50% infection probability in tissue. When exposed to the virus, the probability of becoming infected is a function of the infectious units of virus and mucosal immune state, as shown in Figure 1(C). We used a modified Beta Poisson model calibrated to vaccine challenge infection risk to compute the per virion infectivity of vaccine virus, the rate of mucosal antibody neutralization of virus, and viral interference parameters related to co-infection [3].

The primary motivation for inclusion of intra-host dynamics is to accurately represent observed population heterogeneity in probability of infection, shedding titers and shedding duration and their correlations within a single individual. The model is able to describe how these quantities change over time for a single individual over the course of multiple exposures to virus. The intra-host model is parameterized as described in Behrend *et al.* [3] and makes extensive use of existing polio vaccine challenge data. The ability to represent this heterogeneity and include this correlated data is beyond the reach of a simpler model which does not track individual immune dynamics.

There are two primary structural assumptions of the intra-host model. Firstly we parameterize the priming and boosting of immunity as a linear function of memory immunity which eventually saturates at a maximum level (section 1 equation 1 in File S1). The justification for this can be seen in Figure S1 in File S1which shows the model fit of boosted antibody titer to vaccine challenge data. The saturated linear boosting model provides an excellent fit to the data for each of the 3 serotypes; for type 1 the correlation coefficient is 0.79 with a p value of 0.0006. Secondly, we model acquisition of infection in the absence of viral interference as a Beta Poisson process. This is a standard dose response model used in quantitative risk assessment [22]. The potential advantage of this method over a simpler exponential dose response is its ability to include a non-constant infection rate as the virus in neutralized by antibodies. In the absence of viral interference from co-infection with other virus serotypes, only two parameters are fit in the model, the per virion infectivity of the virus and the rate of neutralization. Fits of the Beta-Poisson model to vaccine challenge data, shown in terms of fraction of individuals in different study groups shedding virus following vaccine challenge are shown in Figure 3 of Behrend *et al.* [3] and reproduced in figure S2 in File S1. It is evident that the model captures the individual heterogeneity in response to vaccine dose. We note that the correlation coefficient between model output and data (0.85, p = 0.0005) was far better than using an individual's dose history alone which had a correlation coefficient of 0.43.

Although polio is endemic in many parts of Northern Nigeria, providing a transmission model for the entirety of Northern Nigeria including detailed migration patterns would introduce an additional layer of model complexity in terms of parameterization and is beyond the scope of this work. Therefore we model a population on the order of the size of the Zaria Local Government Area from which our seroprevalence data is obtained. To reflect the fact that this population is not isolated we include importation of infection at a constant rate of 1 wild polio virus infection per month. We define our elimination threshold as 1 per 300,000 over seven years, as this corresponds to an average of approximately 1 infected individual at any given time. The seven year time horizon was chosen to ensure that the vaccination program was robust with respect to outbreaks from re-importation after temporary cessation of transmission.

The model is calibrated to the baseline of an endemic setting similar to Zaria, Nigeria, between 2008 and 2009 [7]. The task of estimating the poliovirus potential for transmission and prevalence from incidence of paralytic cases is complicated by a low case-to-infection ratio [14], [23] and inconsistent case reporting rate. Therefore, we chose seroprevalence as an indicator to infer transmission parameters from immunity of the overall population and unvaccinated groups. The baseline scenario assumes 30% coverage of RI and that there are six SIAs per year with equal coverage and equally spaced in time, targeted to 0–4 year olds. The level of SIA coverage is ascertained from calibration of the model to Zaria seroprevalence data. SIAs are implemented preventatively, not in response to specific outbreaks. SIAs are given to the entire region considered, and therefore may be considered subnational.

Calibration was performed iteratively via Incremental Mixture Importance Sampling [24], [25]. Four model parameters were calibrated. These are the basic reproduction number of both type-1 vaccine and wild viruses, the historical SIA coverage, and the size of the population which is easily reached by vaccination. Binomial likelihood functions were used to represent the probability of realizing the overall seroprevalence data for age groups 1–4 and 5–9 years (inclusive) as well as the seroprevalence in children aged 1–9 with no reported history of vaccination.

Paralytic case data was not included in the calibration. The probability that our model parameters produced the age stratified seropositivity from the Zaria serosurvey for the overall population and the subset of zero vaccine-dose children is given by the following likelihood function
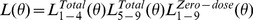
(1)where,
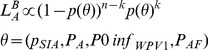
(2)Here, p_SIA_ is the SIA vaccination coverage in the easy to reach group which is taken to be constant over time, P_A_ is the fraction of virions that an individual acquires from exposure to a dose in the community environment, POinf_WPV1_ is the infectivity of WPV1 virus, P_AF_ is the proportion of the population easily accessible by vaccination, A represents the age range in years and B represents the vaccination group, which in this case is either all children or children for whom zero vaccine doses has been reported. Note that vaccination coverage in the hard-to-reach group is computed from p_SIA_ using the relationship outlined in section 2.1 in File S1. *L* is proportional to the probability mass function of the binomial distribution; *p* is the fraction of seropositive individuals in each age group generated from our mathematical model, *n* and *k* are respectively the number of individuals surveyed in the data and seropositive (for each age group). Age-groups are weighted equally in our calibration.

The Zaria serosurvey data, a summary of which appears in Giwa et al. [7] recorded only seroprevalence based on the standard geometric mean titer (GMT) threshold of 8. Therefore, although our simulation model produces more detailed titer distributions, calibration is performed based on seroprevalence using this defined cutoff. It should be noted that as seroprevalence data for unvaccinated individuals between 1 and 9 years old was not further sub-stratified in Giwa et al. [7], it was not possible to completely disaggregate seropositivity by both age and vaccine status at the 1–4 and 5–9 age groups. Therefore we chose the most parsimonious likelihood function composed of the product of the likelihood of the overall age-stratified seropositivity for age groups 1–4 and 5–9 (including vaccinated and unvaccinated children) and the likelihood of the seropositivity in unvaccinated children across the full age range of 1–9 years.

The baseline seroprevalence output from our mathematical model was computed after a 30 year burn in consisting of repeated SIAs of trivalent oral polio vaccine (tOPV) (6 annually) of 0 to 4 year-olds, routine immunization with a fixed coverage of 30%, and introduction of 1 infection per month from outside of our population. We found that 30 years was sufficient for the distribution of humoral antibody titers to stabilize in our model.

## Results

**Figure 2:**
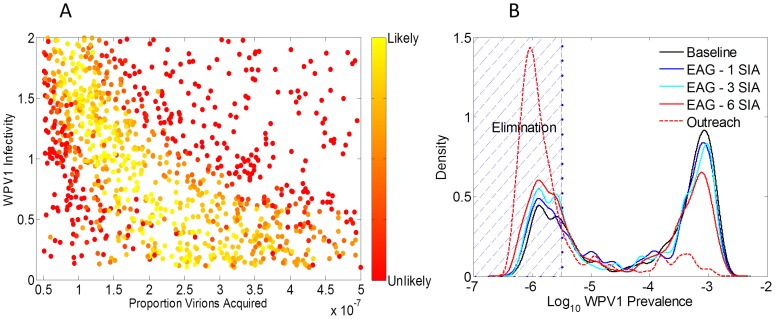
Model calibration and the effect of both expanding age groups and targeting in SIA campaigns. (A) Likelihood of polio infectiousness parameters: relative probability of reproducing the observed seroprevalence data from a sample of the model results conducted in the same manner (number of samples by age) as in the original Zaria serosurvey. Each tick mark represents a two-fold change in likelihood. (**B**) The effect of expanded age group SIA campaigns on elimination: distributions of mean WPV1 prevalence for baseline (calibration), EAG campaigns, and a campaign targeting hard-to-reach groups.

Figure 2(A) shows the likelihood of parameter configurations under the slow mucosal immunity waning rate in terms of the per virion infectivity of the virus, 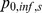 and the fraction of virions an individual acquires when exposed, 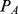. Calibrating to serosurvey data significantly reduced the parameter space of interest by excluding large regions of very low likelihood. This was seen for both slower and faster mucosal immunity waning rates (Figure 2(A) and Figure S6 in File S1). It is of note that inclusion of persistent heterogeneity in vaccination coverage was necessary to effectively reconstruct age-stratified patterns of immunity (see section 2.2B in File S1 and Figure S5 in File S1). Heterogeneity was included in terms of an easy-to-reach and hard-to-reach group (see Figure S4 in File S1 and section 2.1 in File S1). The size of the easy-to-reach group was determined from an initial calibration (see Figure S5 in File S1) and was subsequently then set accordingly to 70% in subsequent calibrations.

To quantify the effect of EAG campaign implementation, we took a posterior distribution sample from the results of our calibration procedure, and compared the overall type-1 wild poliovirus (WPV1) prevalence and age stratified mucosal immunity when the targeted age group was extended to children less than 15 years of age. The probability density of mean prevalence under EAG campaigns is shown in Figure 2B. The cumulative distribution function is shown in Figure S9 in File S1.

**Figure 3:**
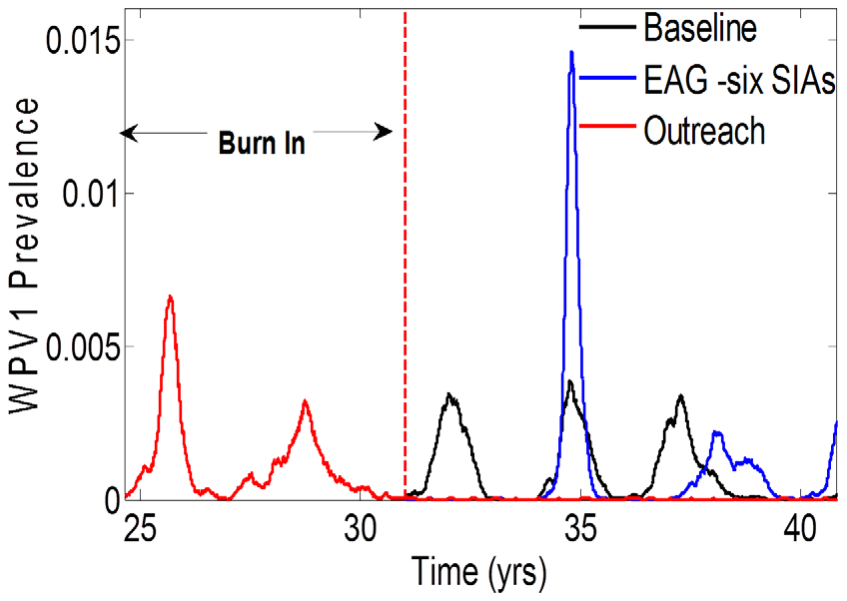
Temporal dynamics of WPV1 prevalence for the most likely set of calibration parameters under baseline conditions, EAG campaigns, and outreach campaigns.

Even under the most optimistic assumptions that EAG campaigns would be employed in 6 SIAs per year and result in a 20% relative increase in overall coverage levels, the probability of elimination increased by 16%. For 3 EAG campaigns this increase was only 8% (Figure S2 in File S1). In contrast, adding 2 outreach campaigns per year with coverage in the hard-to-reach group at only half the baseline coverage of the easy-to-access group resulted in a 55% increase probability of elimination from the baseline. Furthermore, the amount of additional vaccine required in the targeted scenario was less than 1/10^th^ and 1/5^th^ of that for the largest and smallest EAG scenarios respectively. Representative WPV1 temporal dynamics are shown in Figure 3 for baseline, EAG campaigns, and outreach campaigns. Here the calibration parameters are set at their most likely configuration. Recurrent epidemics occur under both EAG and baseline scenarios but not in the targeted outreach campaign.

Though the previous results assume a mucosal antibody half-life life of 7.5 years, for shorter half-lives the results are qualitatively similar. Distributions of mean prevalence assuming a mucosal antibody half-life of 3.5 years are shown in Figures S7 and S8 in File S1. Though the absolute increase in elimination probability is smaller for both the EAG and targeted interventions under the assumption of faster mucosal antibody waning times, the relative impact of the targeted intervention versus the EAG interventions becomes greater. In this case the six SIA EAG campaign results in an 8% (absolute) increase in elimination probability from baseline, whereas the targeted intervention results in a 36% increase.

**Figure 4:**
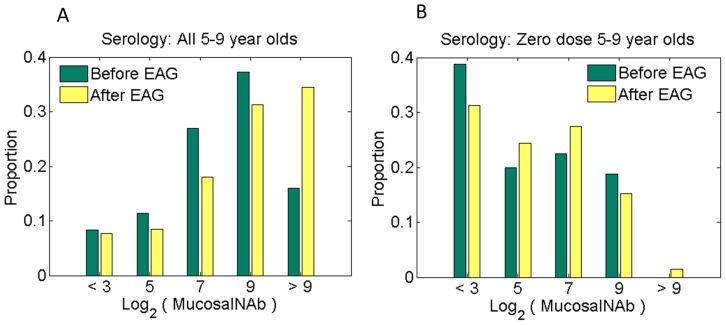
Effect of expanding age groups in SIA campaigns on mucosal immunity. (**A**) Mucosal antibody titer distribution before and after expanded age group campaigns in the overall population (5–9 years) and (**B**) the unvaccinated group (5–9 years) only. log_2_(mucosal antibody titer)<3 represents high susceptibility to infection.

To understand the modest effect of EAG campaigns on elimination, we compared the distribution of mucosal antibody titers before and after EAG implementation. Figures 4 (A) and (B) show this distribution among 5–9 year-olds for our highest likelihood parameter configuration. The EAG campaigns move the distribution of high antibody titers to even higher levels, where there are diminishing returns on reducing the probability of infection and shedding. However, the change in the fraction of individuals with mucosal titers conferring low immunity to infection (log_2_ (titer) <3) is less than 1%. Stratifying these results on children who had never received vaccine (Figure 4(B)) reveals only a small effect on increasing immunity; the proportion of children with low immunity (log_2_ (titer) <3) changes from 69% to 62% in 5–9 year-olds post EAG.

**Figure 5:**
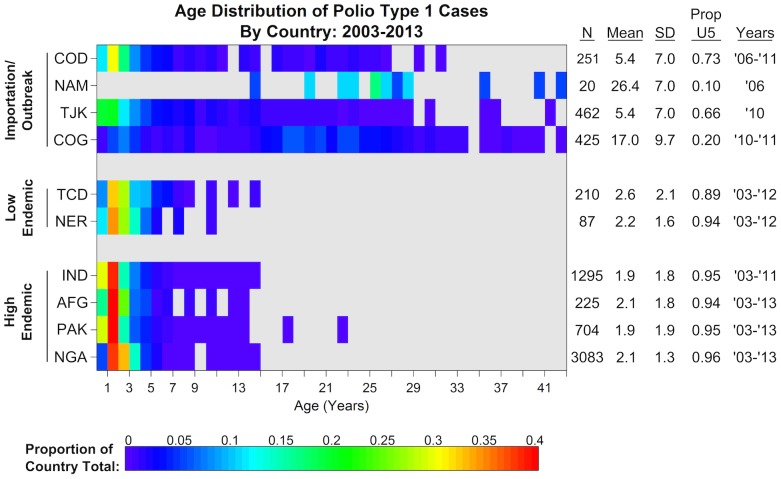
Distribution of cases by age (in years) during previous polio outbreaks for endemic and previously polio-free countries. The number of confirmed type 1 cases, mean age of infection, standard deviation of infection age, proportion of cases under five years old, and duration of case data used are in the table to right. High endemic countries are those that have sustained continuous transmission: India (IND), Afghanistan (AFG), Pakistan (PAK), and Nigeria (NGA). Low endemic areas are those that are exposed periodically to virus due to regular importations: Chad (TCD) and Niger (NER). Importation countries are those that have not reported WPV transmission since at least 2000: Democratic Republic of Congo (COD), Namibia (NAM), Tajikistan (TJK), and Republic of Congo (COG). Information compiled from multiple AFP databases maintained by WHO HQ, regional and country offices.

In contrast, in the absence of recent wild poliovirus circulation, immunity gaps may persist in older age groups due to low historical vaccine coverage. In this case, SIA campaigns with an older target age group could be useful to prevent outbreak and accelerate local elimination efforts. From past polio outbreaks, the distribution of cases by age is useful in showing immunity gaps and transmission dynamics. Figure 5 shows the age distribution of WPV1 cases from countries with no recent WPV circulation (importation/outbreak), countries with low endemic circulation (sustained by Nigeria), and endemic countries. All endemic countries have a low mean age of WPV infection of approximately 2 years. In previously polio-free countries (i.e., no recent WPV circulation), the mean age of infection is decades greater. The difference in age distribution between the importation and endemic scenarios suggests greater immunity gaps in older children in areas with no recent WPV transmission. This is in line with our modeling findings; ongoing WPV circulation can fill in immunity gaps in older children.

**Figure 6:**
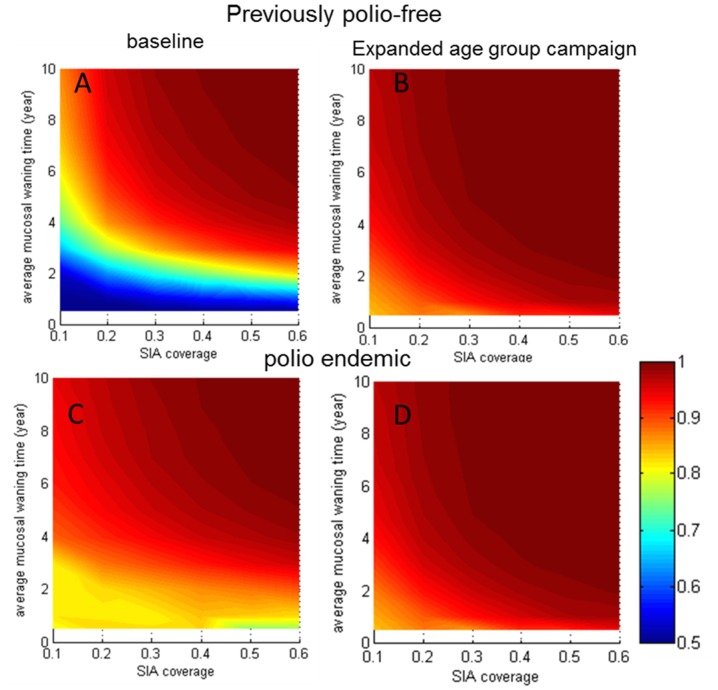
The distribution of mucosal immunity in 5–14 year-olds in EAG campaigns compared with standard campaigns targeting 0–4 year-olds. Average mucosal immunity waning time was varied between 6 months and 10 years (half-life 0.34–7 years), and SIA coverage between 10%–60% per round. We reported the results in fractions of mucosal antibody titer >8, assuming a fixed fraction of acquired viral dose, 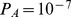. We note that other values of 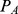 yield similar results: (**A**) no wild poliovirus circulation, baseline; (**B**) no wild poliovirus circulation, expanded age group campaigns; (**C**) with wild poliovirus circulation (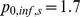), baseline scenario; and (**D**) with wild poliovirus circulation (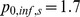), expanded age group campaigns.

To further illustrate both the individual and combined impact of wild poliovirus transmission and EAG campaigns on immunity gaps, we performed simulations on a hypothetical country, and analyzed age dependent immunity both with and without EAG campaigns and wild poliovirus circulation. For both endemic and previously polio-free conditions, we compared the fraction of seropositive older children (aged 5–14 inclusive) with baseline, in the absence of EAG campaigns. As shown in Figures 6 (A) and (B), expanding the age group to reach children under 15-years-old was effective at filling immunity gaps in the absence of previous wild virus circulation. This was true even under the conditions of low historical vaccination coverage and quickly waning mucosal immunity. Additionally, under endemic conditions age gaps in immunity resulting from previous low vaccination disappeared (compare Figures 6A and 6C). In this case EAG campaigns minimally increased the already high immunity (compare Figures 6 C and D). This result agrees with our detailed calibration results showing limited impact of EAG campaigns in polio endemic regions and is consistent with the older ages (>5 years) of observed polio cases from outbreaks in countries with low vaccination coverage and no recent wild-virus circulation.

## Discussion

In endemic countries, expanding the age group in SIA campaigns will not significantly increase overall population immunity or the probability of eradication. Our modeling outcomes show that large immunity gaps in older children cannot be sustained due to frequent boosting from ongoing circulation of wild poliovirus. The age of cases in endemic countries and serology of older children from the pre-vaccination era support our findings. These conclusions are robust for different ranges of mucosal immunity waning rates, vaccine coverage, and virus transmissibility.

In previously polio-free countries, immunity gaps may exist in older children if the virus has been cleared from a region for a long time and there has been historically low vaccine coverage. Therefore, older children may participate in transmission, as observed in several outbreaks where older cases have been reported (Figure 5). In this case, expanding SIAs to older children filled the gap and worked effectively for once-cleared countries to prevent polio outbreaks that might result from importation.

The recent work of Blake *et al.* [26] addressed the utility of expanded group campaigns and the role of older children in transmission during polio outbreaks. By analyzing paralytic case counts from two historical polio outbreaks they concluded that older children were not significant contributors to transmission in the 2010 Tajikistan outbreak, while they did make significant contributions in the 2010 Congo outbreak. In both cases they found that earlier intervention was far superior to expanded age group campaigns.

In comparison, the work presented here answers a related but different question; what is the role of older children in transmission in areas where polio is currently endemically circulating and what benefit would vaccinating older children have in stopping endemic transmission? The key difference between these questions lies in the fact that in an outbreak area polio virus may not have circulated for considerable time, potentially creating immunity age gaps if historical vaccination has been poor.

In an endemic scenario it is important to be able to accurately estimate the contribution of ongoing transmission to population immunity and its subsequent effect on future transmission. To do this we calibrated our model to age-stratified seroprevalence data. We concluded that in this endemic situation, even in the relatively high transmission setting of Zaria Nigeria EAG campaigns will have little effect on polio eradication.

As discussed in the methods section our model is individual based and is able to provide additional realism in terms of heterogeneity in observed probability of infection on exposure to virus as well as duration and intensity of shedding. As such, our results are robust to observed individual heterogeneity in transmission. Furthermore in examining endemic transmission, we directly assessed the role of decaying immunity over time. We concluded that although assuming faster or slower waning influences the calibration of the infectivity parameters as seen by comparing figures 2A and S6 in File S1, it does not affect the conclusion that the utility of EAG campaigns is minimal.

One possible limitation of the study was the assumption of homogeneous mixing between low and high vaccine accessibility groups. In reality this mixing between groups could be setting dependent with much local variation. If we assumed more assortative mixing between groups, EAG campaigns would have an even smaller effect on reducing transmission within the hard-to-reach group where most of the transmission is localized. We did examine the effect of assortative mixing on age-stratified immunity (Figure S10 in in File S1); resulting immunity was both qualitatively and quantitatively similar to the homogenous mixing assumption (see Figure S10 in File S1).

In summary, if expanded age group campaigns can increase coverage among children aged less than 5 years, it becomes important to determine whether this increase would be in groups which have historically been largely unreached by vaccination campaigns. Our results show that in endemic areas, the immunity gap is ‘orthogonal’ to age: reaching chronically missed children is more relevant to achieving eradication than reaching high coverage in older children. As we move closer to polio eradication understanding these heterogeneities in immunity and how to optimally allocate available resources will be critical in realizing our endgame.

## Supporting Information

File S1 **Supporting files. Figures S1–S10. Table S1.**
(DOC)
