# Supplementary material for: Quantifying the Impact of Expanded Age Group Campaigns for Polio Eradication
Source: PLoS One. 2014 Dec 1;9(12):e113538. doi: 10.1371/journal.pone.0113538 (PMC4249973; doi:10.1371/journal.pone.0113538)
Supplement: File S1 — Supporting files. Figures S1–S10. Table S1. (DOC) [file pone.0113538.s001.doc]

**Quantifying the Impact of Expanded Age Group Campaigns for Polio Eradication**

**Supplemental Information**

Bradley G. Wagner, Matthew R. Behrend, Daniel J. Klein, Alexander M. Upfill-Brown, Philip A. Eckhoff, Hao Hu

1. **IDM Disease Modeling Platform**

The polio transmission model is implemented within the more general EMOD modeling framework developed by the Institute for Disease Modeling (IDM). This modular framework, developed in C++, allows for construction of individual based disease models. The model is specified in multiple levels. First, there is a demographic level which accounts for population distribution in terms of age, gender and other risk factors and individual properties. A second level accounts for population level disease transmission aspects such as mixing between different groups. A third level includes intrahost modules which describe individual immunity and infectiousness. The EMOD framework has been used in modeling both malaria and HIV ^1, 2^. The immunity and infectiousness aspects which comprise the within-host polio modules are described in detail in Behrend et al. ^3^ and in the following sections.

The polio model represents low-level processes of disease transmission. The model is not a set of differential equations, but rather a collection of processes and events, which interact through various mechanisms, each with its own mathematical relationship. The combination of inputs and vaccine interventions produce a diversity of individual immune states that can be captured well in individual-based models.

***1.1 Intrahost Model***

The intrahost model includes immunity, infection, and other states of the individual. These model the effects of age, maternally-derived antibodies, time-varying immunity to infection and to paralysis, poliovirus serotype, poliovirus evolution, immune response to challenge, internal viral load, demographic risk factors, and host factors in OPV efficacy. Outputs from the intrahost model include immune state, paralysis, and shedding in units of tissue culture infectious dose (TCID_50_).

**1.1A Immunity and Protection against Infection**

Live virus infection (OPV / WPV) target different immune compartments. Mucosal and humoral immunity to poliovirus are usually loosely defined as immunity to infection and disease, respectively. To model the effects of OPV, we track mucosal neutralizing antibodies and humoral neutralizing antibodies separately. We take serum neutralizing antibodies as a direct measurement of humoral immunity. Mucosal immunity is typically measured as resistance to shedding at challenge. We assume that mucosal immunity can be similarly represented by an unobserved neutralizing antibody titer that is a predictor for resistance to challenge. This mucosal titer is impacted with different weight by OPV immunization.

In the model, we quantify immunity by neutralizing antibody titer to poliovirus. Neutralizing antibody titer is variable for any given dose history ^3^. The relationship between antibody titer and probability of shedding is complicated by factors that include waning immunity, memory immunity, gut integrity, maternal antibodies, intertype viral interference ^4^, and the type of IPV/OPV vaccination history. Interpretation of studies is confounded by these factors. We conducted a meta-analysis of vaccine studies to parameterize a poliovirus immunity model ^3^ that is contained in each individual of this disease transmission model.

**1.1B Immune Response to Infection**

Immune response to an infection depends more upon the state of priming (memory immunity) than the present antibody titer. For example, recipients are sometimes seronegative following the first dose but exhibit boosting upon subsequent doses. We use memory immunity to calculate the boosting effect of subsequent exposures to antigen. However, Abbink et al. ^5^ found that memory immunity in seronegative elderly subjects did not protect against infection upon mOPV challenge. Therefore, immunity to infection is calculated from present-day neutralizing antibody titers for the individual.

Upon the first live infection, we determine the individual’s NAb titer, *M_firstLive,s_*, from a distribution of titers observed at first exposure in analysis of vaccine studies ^3^. If the individual has no memory immunity (including ‘priming’) to the infecting serotype, then the infection is effectively the first antigenic exposure. This is not necessarily the first vaccine dose because prior doses may have failed to take.

Once priming has been established for a serotype (
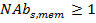
), subsequent exposures to that antigen use immune boosting equations. The boosting response is treated as a linear increase in logarithmic titer from the memory immune state. The parameters *b_boost_* and *Log_2_NAb_max_* define the infection immune response along with a stochastic component having a standard deviation *σ_boost_* and a unit-normal random variable *r_n_*.


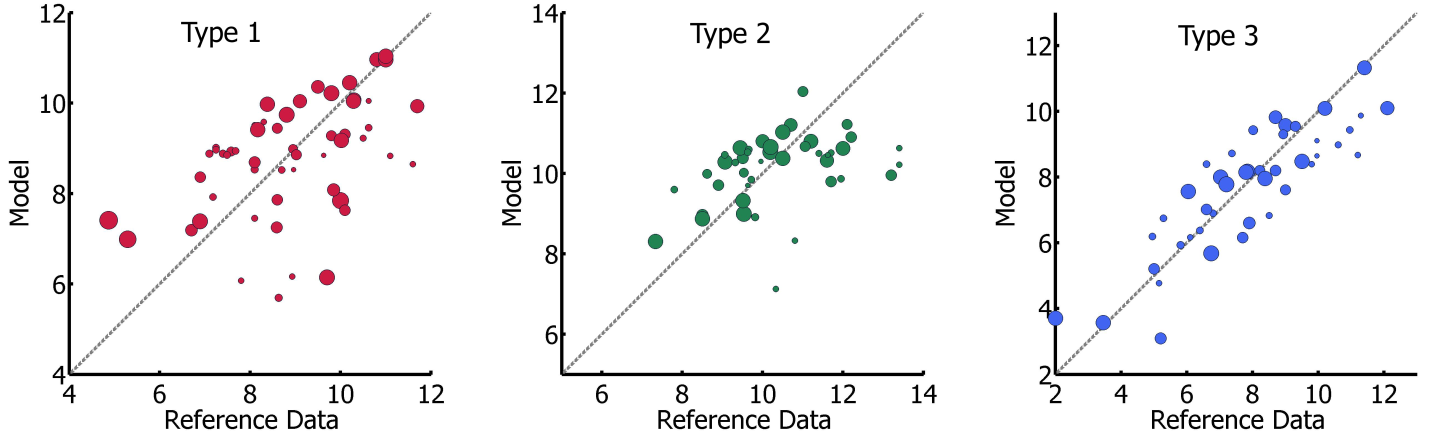


**Figure S1**. OPV boosting model fit to data. Axes are post-vaccine serum titer in log_2_GMT. From left to right, red, green, blue plots are serotypes 1, 2, 3, respectively. Correlation coefficients are 0.79, 0.42, 0.85; p = 0.0006, 0.003, 0.0007; slope 0.71, 0.27, 0.68; serotypes 1, 2, 3 respectively.

The following equation sets for neutralizing antibody titer consider the memory *NAb_s,mem_* of each immune compartment in calculating the new titer of serotype *s* at time *t*. The parameters for priming and boosting are specific to the poliovirus serotype and to the form of the antigen.


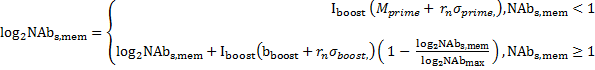
 *(1)*

The immunogenicity of the antigen is unity (
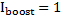
) for live virus infections.

**1.1C Acquisition Immunity**

****( much of the content of section 1.1C appeared originally in Behrend et al. 2013 and is included in a modified form here for completeness)^1^**

**OPV**

Acquisition immunity is described by a separate component of parameters that relate immune state to the probability of infection. The three Sabin strains of poliovirus each have a specific infectivity parameter, P0inf_s_. Neutralizing antibody reduces the probability of infection from the challenge dose. Three more parameters, ViralIntf_s_ where *s* is one of serotype 1, 2 or 3, measure viral interference in multivalent doses of OPV. Another parameter, τ_Ab_, is the time constant for neutralization of the virus dose.

Single strain acquisition (probability of infection conditioned on absence of viral interference) is taken as a beta-Poisson process at a distributed rate R having a probability of 1 – exp(-R) for a nonzero number of events ^6^. The probability of infection by serotype s with PVDose_s_ quantity of TCID_50_ poliovirus is,


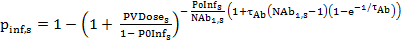
 *(2)*

Neutralizing antibodies NAb_1,s_ are the model’s representation of mucosal immunity and are computed from the immune priming and boosting equations for all previous vaccine doses before the present vaccine challenge. This quantity can be interpreted as the equivalent serum titer that would yield the same mucosal immunity in an OPV-only-immunized individual. Probability of infection by each serotype s from a multivalent exposure is the product of the single strain probability and non-interference by other Sabin strains.


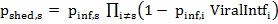
 *(3)*

Viral interference is discussed in more detail in SI section 1.1d.

The mucosal immunity parameters were fit to fecal shedding of poliovirus, and thus reflect intestinal immunity as opposed to pharyngeal immunity. Throughout this work we make the simplifying assumption that within Northern Nigeria, infection occurs through the fecal-oral route. The intestinal immunity to infection was inferred from a combination of serum neutralizing titer and dose history. For OPV recipients, the number of doses or the serum neutralizing titer can indicate intestinal immunity. The model fit of serum neutralizing titer mediated vaccine efficacy to OPV vaccine challenge data is shown in figure S2.


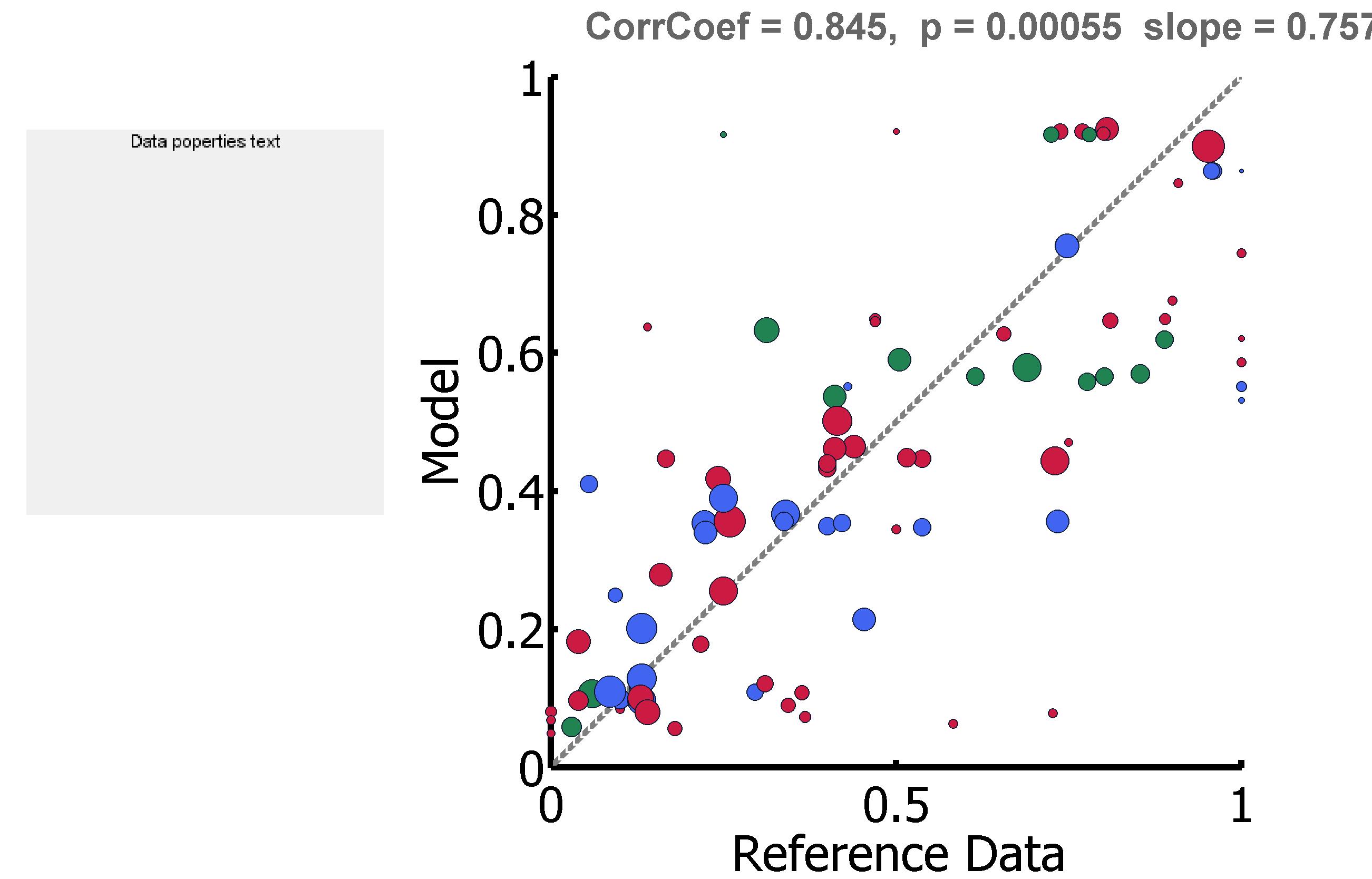


**Figure S2**. Model fit for antibody-mediated vaccine efficacy against infection. Both axes are the fraction of subjects shedding virus upon challenge in each study group. Studies shown are OPV only vaccine schedules. Red, green, and blue markers are serotypes 1, 2, and 3, respectively. Correlation coefficient 0.85, p = 0.00055, slope 0.76. Identity line (dashed) represents a perfect model.

**Community Acquisition**

For wild or vaccine virus acquired from the community (i.e., transmission as opposed to vaccination) p_inf,s_ is computed analogously as that for OPV vaccination with one additional factor to account for environmental factors related to virus acquisition. The probability of infection is given by:

$p_{\inf,s}=1-\left(1+d_{A}\right)^{\frac{-p0\inf,s}{NAb_{1,s}}\left(1+\tau_{AB}\left(NAb_{1,s}-1\right)\left(1-e^{-1/\tau_{AB}}\right)\right)}$. (4)

Here, d_A_ is the dose of virus acquired and is given by

$d_{A}=d*P_{A}$ (5)

where d is the maximum dose an individual may be exposed to in the environment and P_A_  is the proportion of virions acquired, representing the fact that an individual is only exposed to a fraction of viruses in the environment. Viral interference occurs exactly as described previously in equation 3.

**1.1D Viral Interference**

Simultaneous exposure to multiple poliovirus types allows for competition among them for replication sites in the gut. Trivalent OPV is effective at conferring immunity to type 2, but individuals often do not seroconvert to type 1 or type 3 on the first dose. Viral interference from type 2 prevents the other serotypes from productively infecting the host ^4^. This is the basis for a ‘balanced’ formulation of tOPV containing three strains in 10:1:6 proportions. However, this competitive behavior is not exclusive to type 2. Our analysis indicates that a type 3 infection may also interfere with vaccine take for the other Sabin strains; however the type 3 infection has less opportunity to do so because of its lower infectivity. Pre-existing immunity to each serotype of poliovirus is also a factor in vaccine take.

Secondary spread of vaccine strains is necessarily part of the process. In a dose of tOPV, not all serotypes are shed, primarily because of immunity, viral interference, and host factors ^7^. In a multivalent vaccine dose (tOPV or bOPV), we calculate the probability of infection *P_inf,s_* in each individual for serotype *s* as if the virus exposures were independent. We then calculate the probability of vaccine take for each serotype *s* discounting by the probability of viral interference from infections produced by the other serotypes (as shown in equation 3). Viral interference is not limited to vaccine doses, but also considers VDPV and WPV infections already established in the host. Individuals may experience superinfections of differing serotypes. Estimates of the magnitude of viral interference for each serotype for are given in Table S1 as computed in Behrend et al. ^3^.

**1.1D Maternal Immunity**

Maternal antibodies can confer protection against infection and paralysis during their first months. Maternal immunity decay is modeled for all newborns in the simulation as:


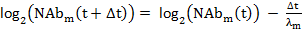
 *(6)*

Log(NAbm) for newborns is given by a truncated normal distribution with a mean of 6 and standard deviation of 3. Here, truncation refers to the removal of data points beyond 2 standard deviations from the mean. The half-life (*λ_m_*) of maternal antibody is obtained by linear regression of the *log_2_NAb* titer from serology pairs in infants before exposure to vaccination. Regression of the existing maternal antibody waning studies gave a half-life of 22 ± 5 days (Figure S3 and Table S1.


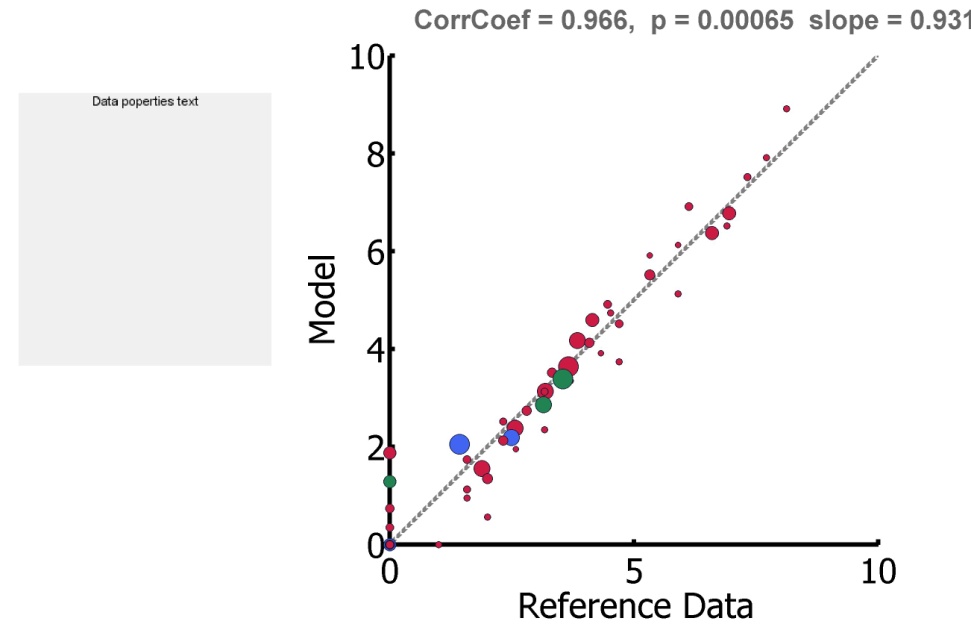


**Figure S3**. Maternal antibody model correlation to data. Measurement on both axes is log_2_GMT after the waning period between serology pairs. Red, green, blue markers are serotypes 1, 2, 3, respectively.

**1.1E Waning Immunity**

For humoral waning immunity, the fast-waning process occurs at rate
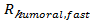
. A slower component decays at rate
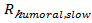
. The duration of the fast waning process is set at two years, reflecting data which shows strong decay in geometric mean antibody titers over the first two years followed by stabilization ^8^. The fast waning half-life is set at 1.7 years which is consistent with the range observed from data in the first two years after boosting ^9-12^. The slow waning rate is parameterized from antibody titers of two cohorts in Sweden who received IPV ^13^. It is notable that Sweden had no wild-virus circulation at this time and killed IPV vaccine was in use. Therefore differences in antibody titer are not complicated by boosting from natural or vaccine virus infection. Also, these cohorts represented individuals who had received their last booster between 6 and 10 years ago. We set the half-life of the slow waning process to be 4.0 years ^8, 13^. The equation for humoral antibody waning is given below where *t* represents the time since the last boost.

$\Delta NAb_{humoral,s}=NAb_{humoral,s}\Delta t(H(t-2)R_{humoral,fast}+R_{humoral,slow}(1-H(t-2)))$ (7)

where H(t-2) is the standard Heavyside function shifted by two years.

For mucosal waning immunity, we assume that antibodies wane in an exponential form with a single rate
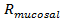
. Since studies are needed to better characterize long-term (many years) waning ^5, 8, 13-18^, particularly with regard to shedding upon challenge, we selected two scenarios with
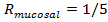
years (half-life 3.5 years) and
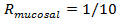
years (half-life 7.5 years). Waning of mucosal antibodies is given by the following formula:


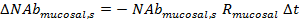
(8)

**1.1F Infection Duration**

Infection duration *D_I,s_* is about 6-8 weeks in fully-susceptible individuals. Prior exposure to wild virus, OPV, or IPV will reduce the duration of shedding ^19^. Pre-challenge immune state discounts shedding duration from a maximum duration *D_I,max_* by the log of mucosal immunity *Log_2_NAb_muc,s_* and a parameter *k_DI_*. Duration is assumed to be stochastic with a normal distribution of relative standard deviation *σ_DI_*


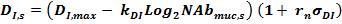
 *(9)*

Poliovirus is excreted from the pharynx and in feces. Pharyngeal excretion is limited compared to fecal excretion, but may play an important role in some transmission settings. For the setting of Northern Nigeria we made the simplifying assumption that transmission is fecal-oral.

We have assumed that viral load is constant over the duration of infection and quantify viral particles using the number shed in feces. In reality, subjects shed peak titers within a couple of days from inoculation with OPV ^20-23^. Titer in individual subjects actually varies substantially from day to day, may decline and then rise again to peak levels. The time-varying excretion titer is relevant to the generation time between infections. The model assumes that fecal excretions are spread uniformly over the infectious period. This is known to be untrue ^24^. Dynamic shedding titer will be explored in further analysis of the data.

**1.1G Shed Virus Titer**

Neutralizing antibodies are assumed to reduce the shedding titer (S_1,s_) linearly on a log-log chart with slope –*k_shed_*


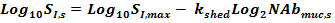
 *(10)*

Less than 1% of poliovirus infections are clinically manifest as paralytic cases in fully-susceptible hosts. Due to the importance of case reporting in the epidemiology of polio, we consider the differential immunity to infection and to paralysis. Seroconverted individuals are immune to paralysis but may still become infected and participate in chains of transmission. Estimated case-to-infection ratios in immunized populations ^25^ can range from 1:1000 to 1:10,000. In Madura, for example, more than 100,000 infections were likely during a cVDPV outbreak of 56 paralytic cases ^25^.

**2. Transmission Model**

Virus transmission equations mediate infectious contact between individuals. Individuals in close proximity are assumed to shed and acquire virus from a homogeneously mixed contact pool. Virus shed is summed from all infectious individuals, separately counting each serotype and genome in the mixing pools. The total virus is normalized by population for density-independent contact patterns. Individuals in contact with a mixing pool acquire a fraction of the total virus, according to their contact strength. If exposure results in infection, the identity of the virus serotype and genome are drawn from the distribution in the pool. Not all strains are equally infectious; transmissibility is dependent upon serotype.

The model takes all strains of WPV into account during simulations. Infectious individuals shed a quantity of virus in units of tissue culture infectious dose (TCID_50_) tagged with a strain identity. Strain identity consists of a virus type identifier. The types of viruses are WPV1, WPV2, WPV3, VDPV1, VDPV2, and VDPV3.

***2.1 Parameterization***

The mathematical model was parameterized using Zaria Nigeria type demographics with a population of 300,000 individuals. The model allows for heterogeneity in accessibility in vaccination by specification of vaccine-accessible and vaccine-inaccessible groups. The relative accessibility of the two groups is specified by a power law, determined by the specified overall coverage in the population. The relationship between overall coverage and coverage in the accessible and inaccessible groups is shown in Figure S4.

**
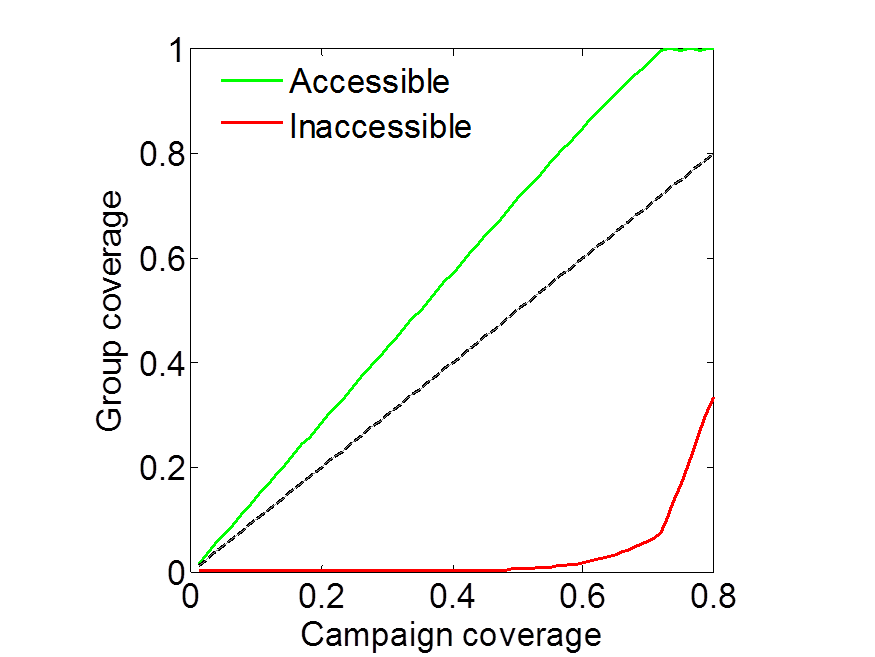
**

**Figure S4:** Fraction vaccinated in vaccine-inaccessible (red) and vaccine-accessible (green) groups as a function of population level SIA campaign coverage (dashed black line). Here the size of the vaccine-accessible group is set at 70%, the most likely value obtained from our model calibration (Figure S5). Coverage in the vaccine-accessible group follows a power law with respect to the population level coverage. The accessibility power is set at 8 throughout our simulations.

We assume homogeneous mixing between age groups, both for parsimony and to realize the maximal effect of vaccine virus shedding from older to younger children in our EAG vaccination campaigns. For the same reason we allow mixing between the vaccine-inaccessible and vaccine-accessible groups to be homogeneous.

As previously stated, we investigated cases of relatively fast and slow mucosal waning, consistent with previously estimated ranges of mucosal waning rates. The model was re-calibrated for each mucosal waning case. The calibration procedure is discussed in the following section.

***2.2 Calibration***

**2.2B Data and Results**

The serosurvey data used in the calibration was collected in between 2008 and 2009 in Zaria Nigeria ^26^. This cross-sectional, community based survey consisted of healthy children between the ages of 1 and 10 years. The study employed a multistage sampling technique, proceeding firstly from the two governments comprising metro-Zaria, Saria Gabon and Zaria. The next stages consisted of randomly selecting two wards from each of these local governments, and finally one settlement from each ward. At this level a systematic random household sample was used to obtain one child per household until reaching the desired sample size. For each child in the survey, antibody titers were determined according to WHO guidelines by a micro neutralization assay.

Figure S5 shows the posterior probability density and cumulative distribution for the size of the vaccine-accessible population. Here the mucosal antibody half-life is set at 7.5 years. From the density plot we see that it is far more likely that the size of the vaccine accessible group lies between 65% and 75% of the total population. This underscores the necessity of including heterogeneity in vaccine accessibility in explaining age-stratified immunity in the population.

Calibration results assuming a mucosal antibody half-life of 3.5 years are shown in Figure S6. The likelihood of the parameter space (in terms of WPV1 specific infectivity and proportion of virions acquired) is shown on a logarithmic scale identical to that in Figure 2A in the main text. Each tick of the color bar represents a twofold change in likelihood. We see that the maximum likelihood achieved is indistinguishable from that shown in Figure 2A. Furthermore we see a similar tradeoff between WPV1 specific infectivity and the proportion of virions acquired, resulting in a band of high-likelihood. The location of the band is at lower values than that seen for the mucosal antibody half-life of 7.5 years. This reflects the increased shedding of virus from older-age groups due to a quicker loss of (mucosal) immunity.

**Figure S5:** Posterior probability density (red) and cumulative distribution function (black) for the size of the vaccine accessible group assuming a mucosal antibody half-life of 7.5 years A range of significantly higher likelihood exists from approximately 65% to 75%.

**Figure S6:** Likelihood of parameter configurations in terms of WPV1 specific infectivity

and Proportion of virions acquired, assuming a mucosal antibody half-life of 3.5 years. Likelihood is shown on a logarithmic scale with each tick representing a two-fold change.

**3. Additional Results**

***3.2 Probability of Elimination***

The posterior distribution of WPV1 prevalence assuming the shorter mucosal antibody-half-life of 3.5 years is shown in Figures S7 (compare with Figure 2B in the main text). The probability densities are shown under our baseline calibration, EAG campaign (6 EAG SIAs yearly), and an outreach campaign. For the outreach campaign two SIAs per year have increased coverage in the inaccessible group at half the coverage level of that in the accessible group. Results are analogous to those seen for the mucosal antibody half-life of 7.5 years (Figure 2B in main text); the outreach campaign dramatically reduces prevalence to eradication levels, whereas EAG results in only a small improvement in eradication prospects. Cumulative distribution functions for WPV1 prevalence, for both the 3.5 and 7.5 mucosal antibody half-lives are shown in Figures S8 and S9.


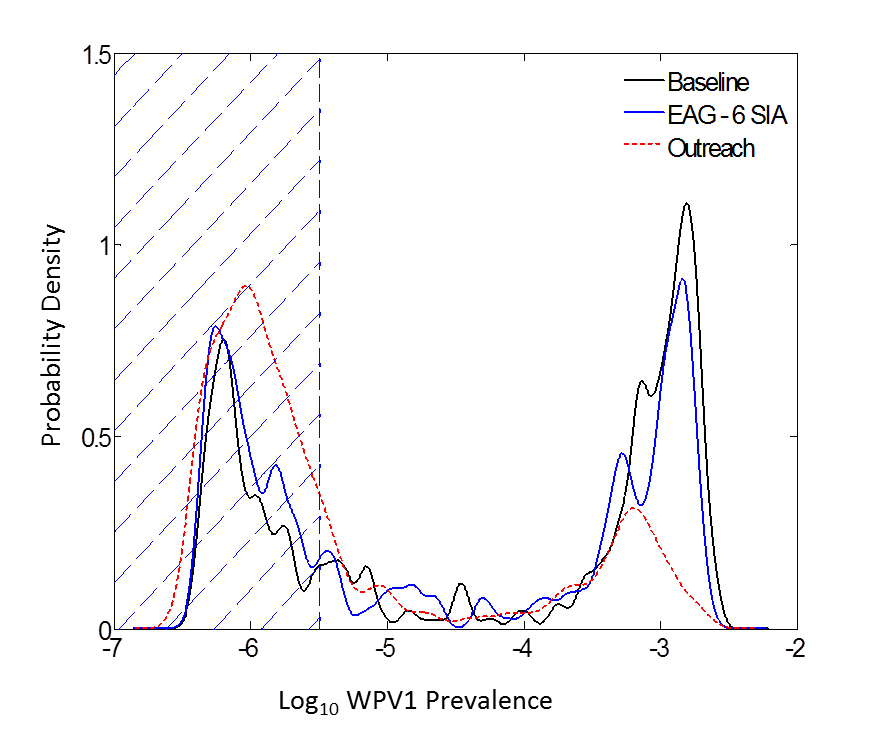


**Figure S7**: Probability density function for Log_10_ mean prevalence of type-1 wild poliovirus, assuming a mucosal antibody half-life of 3.5 years.

**Figure S8:** Cumulative distribution function for Log_10_ mean prevalence of type-1 wild poliovirus, assuming a mucosal antibody half-life of 3.5 years.

**Figure S9:** Cumulative distribution function for Log_10_ mean prevalence of type-1 wild poliovirus, assuming a mucosal antibody half-life of 7.5 years.

***3.3 Contact Patterns and Age Stratified Immunity***

A critical reason for the limited impact of EAG campaigns in endemic settings is their inability to reach unimmunized clusters. However, it is important to note that immunity gaps between unvaccinated and vaccinated individuals decrease over time due to secondary immunization and wild virus circulation. As shown in Figure S10 this effect is robust with respect to assumptions of contact patterns between age groups. This increased immunity with age is consistent with several seroprevalence studies from previously endemic countries. In these studies, even when older children and adults remained unvaccinated in the pre-vaccine era, no significant immunity gaps were found. In endemic settings, expanding vaccination to older age groups would likely reach children who are already immunized.

**Figure S10:** Distribution of seropositivity rates stratified by age showing the effect of boosting from wild poliovirus in older children (most likely baseline scenario, mucosal antibody half-life of 7.5 years). Both homogeneous contact mixing between age groups and age-assortative mixing yield similar immunity patterns. For age-assortative mixing we assume individuals are twice as likely to be in contact with their own group. Age groups considered were 0-4, 4-9 9-14 and 15 years of age and older.

**Table S1: Polio Model parameters**

| **Parameter** | **Description** | **Value** | **Units** | **References/ additional info** |
| --- | --- | --- | --- | --- |
| M_prime,s_ | Mean immunity priming increase | s=1, 5.4  s=2, 6.17  s=3, 3.11 | Log2(GMT) | ^3^ and refs therein |
| σ_prime,s_ | Standard deviation of immunity priming increase | s =1, 2.2  s=2, 2.5  s=3, 2.7 | Log2(GMT) | ^3^ and refs therein |
| B_boost_ | Mean immunity boosting increase | s =1, 5.33  s = 2, 6.17  s = 3 | Log2(GMT) | ^3^ and refs therein |
| σ_boost_ | Standard deviation of immunity boosting increase | s = 1, 0.5  s = 2, 2.5  s = 3, 0.4 | Log2(GMT) | ^3^ and refs therein |
| Log2NAbmax_s_ | Saturation level of geometric mean titer for serotype s | i=1, 10.7  i=2, 11.7  i=3, 15 | Log2 (GMT) | ^3^ and refs therein |
| k_shed_ | Immunity reduction in shedding intensity | 0.057 | Log10(shed titer)/log2(GMT) | 23 |
| λ_m_ | Maternal antibody half life | 22 | days | 4, 27 |
| D_1,max_ | Maximum infection duration | 25 | days | 23 |
| K_di_ | Immunity reduction in shed duration | 0.057 | Log10(days)/log2(GMT) | 23 |
| τ_AB_ | Neutralization factor for poliovirus by antibodies | 0.038 | Dimensionless | ^3^ and refs therein |
| Viralintf_i_ | Viral interference type 1 | s=1, 0  s=2, 0.16  s=3, 0.14 | Dimensionless | ^3^ and refs therein |
| P0Inf_1_ | VPV1 Virus Specific Infectivity | 0.17 | Dimensionless | ^3^ and refs therein |
| P0Inf_2_ | VPV2 Virus Specific Infectivity | 0.29 | Dimensionless | ^3^ and refs therein |
| P0inf_3_ | VPV3 Virus Specific Infectivity | 0.13 | Dimensionless | ^3^ and refs therein |
| P0inf_WPV1_ | WPV1 virus specific infectivity | 0-2 | Dimensionless | Calibrated |
| PA | Proportion of virions acquired from community virus dose (transmission) | 5e-8 – 5e-7 | Dimensionless | Calibrated |
| p_SIA_ | SIA vaccination coverage in hard to reach group | 0-1 | Dimensionless | Calibrated |
| P_AF_ | Proportion of population easily accessible by vaccination | 0.6-1 | Dimensionless | Calibrated |
| R_mucosal_ | Waning rate of mucosal antibodies | 0.1, 0.2 | Years^-1^ | Low, High |
| Rhumoral_fast | Fast waning rate of humoral antibodies | 0.4 | Years^-1^ | 14 |
| R_humoral,slow_ | Slow waning rate of humoral antibodies | 0.17 | Years^-1^ | 8, 13 |

**References:**

1. Bershteyn A, Klein DJ, Eckhoff PA. Age-dependent partnering and the HIV transmission chain: a microsimulation analysis. J R Soc Interface. 2013;10(88):20136013.

2. Wenger EA, Eckhoff PA. A mathematical model of the impact of present and future malaria vaccines. Malaria J. 2013;12(126).

3. Behrend M, Hu H, Nigmatulina K, Eckhoff P. A quantitative survey of the literature on poliovirus infection and immunity. Int J Infect Dis. 2013;18:4-13.

4. Galindo M. The Cuba IPV Collaborative Study Group. Randomized placebo controlled trial of inactivated poliovirus vaccine in Cuba. N Engl J Med. 2007;356:1536-44.

5. Abbink F, Buisman AM, Doornbos G, Woldman J, Kimman Tjeerd G, Conyn-van Spaendonck MAE. Poliovirus-specific memory immunity in seronegative elderly people does not protect against virus excretion. The Journal of Infectious Diseases. 2005;191:990-9.

6. Teunis PFM, van der Heijden OG, van derGiessen JWB, Havelaar AH. The dose-response relation in human volunteers for gastro-intestinal pathogens. Rijksinstituut voor Volksgezondheid en Milieu (Netherlands). 1996;Report no. 284550 002.

7. Serazin AC, Shackelton LA, Wilson C, Bhan MK. Improving the performance of enteric vaccines in the developing world. Nature Immunology. 2010;11(9):769-73.

8. Bottiger M. Polio immunity to killed vaccine: an 18-year follow-up. Vaccine. 1990;8:443-45.

9. Salk D, van Wezel AL, Salk J. Induction of long-term immunity to paralytic poliomyelitis by used of non-infectious vaccine. The Lancet. 1984;2(8415):1317-21.

10. Faden H, Modlin JF, Thoms ML, McBean AM, Ferdon MB, Ogra PL. Evaluation of Immunization with Live Attenuated and Enhanced-Potency Inactivated Trivalent Poliovirus Vaccines in Childhood: Systemic and Local Immune Responses. The Journal of Infectious Diseases. 1990;162(6):1291-7.

11. Swartz TA, Ben-Porath E, Kanaaneh H, Leitner L, Goldblum N. Comparison of Inactivated Poliovirus Vaccine and Oral Poliovirus Vaccine Programs in Israel. Reviews of Infectious Diseases. 1984;6(S2):S556-S61.

12. Nishio O, Ishihara Y, Sakae K, Nonomura Y, Kuno A, Yasukawa S*, et al.* The trend of acquired immunity with live poliovirus vaccine and the effect of revaccination: follow-up of vaccinees for ten years. Journal of Biological Standardization. 1984;12(1):1-10.

13. Bottiger M. Long-Term Immunity following Vaccination with Killed Poliovirus Vaccine in Sweden, a Country with No Circulating Poliovirus. Reviews of Infectious Diseases. 1984;6(S2):S548-S51.

14. Faden H, Duffy L, Sun M, Shuff C. Long-Term Immunity to Poliovirus in Children Immunized with Live Attenuated and Enhanced-Potency Inactivated Trivalent Poliovirus Vaccines. The Journal of Infectious Diseases. 1993;168(2):452-4.

15. Verlinde JD, Wilterdink JB. A small-scale trial on vaccination and revaccination with live attenuated polioviruses in the Netherlands. First International Conference on Live Poliovirus Vaccines. 1959:355-66.

16. Triassi M, Ribera G, Barruffo L, Barbone S, Medda E, Grandolfo ME. Persistence of Immunity to Poliomyelitis among a Southern Population That Received Four Doses of OPV 5 to over 15 Years before. European Journal of Epidemiology. 1966;12(1):5-8.

17. Nates SV, Martinez LC, Barril PA, Ferreyra LJ, Giordano MO, Masachessi G*, et al.* Long-Lasting Poliovirus-Neutralizing Antibodies among Argentinean Population Immunized with Four Or Five Oral Polio Vaccine Doses 1 Month to 19 Years Previously. Viral Immunology. 2007;20(1):3-10.

18. Green MS, Handsher R, Cohen D, Melnick JL, Slepon R, Mendelsohn E*, et al.* Age differences in immunity against wild and vaccine strains of poliovirus prior to the 1988 outbreak in Israel and response to booster immunization. Vaccine. 1993;11(1):75-81.

19. Ghendon YZ, Sanakoyeva II. Comparison of the resistance of the intestinal tract to poliomyelitis virus (Sabin's strains) in persons after naturally and experimentally acquired immunity. Acta Virologica. 1961;5:265-73.

20. Dick GWA, Dane DS, McAlister J, Nelson R, Field CMB. Vaccination against poliommyelitis with live virus vaccines 7. Effect Of Previous Salk Vaccination On Virus Excretion. British Medical Journal. 1961;2(5247):266-9.

21. Davis DC, Lipson MJ, Carver DH, Melnick JL, Robbins FC. The degree and duration of poliomyelitis virus excretion among vaccinated household contacts of clinical cases of poliomyelitis. Pediatrics. 1958;22(1):33-40.

22. Piirainen L, Stenvik M, Roivainen M, Eskola J, Beuvery EC, Hovi T. Randomised, controlled trial with the trypsin-modifed inactivated poliovirus vaccine: assessment of intestinal immunity with live challenge virus. Vaccine. 1999;17:1084-90.

23. Alexander JP, Gary HE, Pallansch MA. Duration of poliovirus excretion and its implications for acute flaccid paralysis surveillance: a review of the literature. J Infect Dis. 1997;175(S1):S176-82.

24. Ogra PL, Karzon DT. Poliovirus antibody response in serum and nasal secretions following intranasal inoculation with inactivated poliovaccine. The Journal of Immunology. 1969;102(1):15-23.

25. Wringe A, Fine PEM, Sutter RW, Kew OM. Estimating the extent of vaccine-derived poliovirus infection. PLoS ONE. 2008;3(10):e3433.

26. Giwa F, Olayinka A, Ogunshola F. Seroprevalence of poliovirus antibodies amongst children in Zaria, Northern Nigeria. Vaccine. 2012; 30(48):65759-65.

27. Gelfand H, Fox J, Leblanc D, Elveback L. Studies on the developmen of natural immunity to poliomyelitis in Louisiana V. Passive transfer of polio antibody from mother to fetus and natural decline and disappearance of antibody in the infant. J Immunol. 1960;85:46-55.
